# Time to positivity: a useful parameter to evaluate intensive care unit blood stream infections?

**DOI:** 10.5935/0103-507X.20200049

**Published:** 2020

**Authors:** Suellen Gavronski, Keite da Silva Nogueira

**Affiliations:** 1 Departamento de Microbiologia, Hospital de Clínicas, Universidade Federal do Paraná - Curitiba (PR), Brasil.

**Dear editor,**

Bloodstream infections (BSI) are frequent and serious complications in intensive care units (ICU) and are associated with high morbidity and mortality rates, increased hospital stay and healthcare-related costs.^(1-3)^

In general, blood culture is the most important laboratory resource for the diagnosis and investigation of BSI; in addition, blood cultures also provide information regarding time to positivity (TTP), which can be used to predict prognosis, allow the evaluation of the efficacy of current antimicrobial therapies and are important to evaluate bacterial load and differentiate real infections from contaminants.^(4,5)^ However, the use of TTP to evaluate blood culture results is still questioned.

This retrospective study aimed to analyze the importance of TTP of microorganisms related to BSI in patients admitted to the ICU of a tertiary hospital in Curitiba from June 2013 to May 2018. The study was approved by the ethics committee of research involving humans (reference number 067486/2016; approval letter dated November 2016).

Blood cultures were obtained from patients with suspected BSI and were incubated on a BD BACTEC™ FX^®^ automated system. Positive blood culture isolates were identified using VITEK 2^®^ automated system (bioMérieux, Durham, North Carolina) and standardized methodologies.^(6)^ Repeated monomicrobial episodes of bacteremia with the same pathogen isolated from the same patient within a month accounted for one blood culture. Polymicrobial cultures were excluded from the study. Time to positivity was recorded for each positive sample. When more than one culture bottle was positive, the first TTP was recorded.

Coagulase-negative *staphylococci* (CNS) were classified according to the number of positive bottles: one (CNS +) was considered a contaminant, and two or more (CNS ++) were considered true BSIs. All statistical analyses were performed using SPSS version 20.0, and a p value < 0.05 was considered statistically significant.

In the 5-year study period, 5,425 blood cultures were collected from ICU; 107 were polymicrobial and 968 were positive for one microorganism, resulting in a positivity rate of 19%. Among the analyzed cultures, in 194 were identified contaminant CNS (contaminant rate 3.5%), resulting in 774 true positive blood cultures.

Gram-positive pathogens were the most frequently isolated pathogens in the ICU-BSI samples (n = 502, 64.8%), followed by gram-negative pathogens (n = 214, 27.7%), fungi (n = 56, 7.3%) and acid-alcohol-resistant bacillus (n = 2, 0.2%). Coagulase-negative *staphylococci* were the most prevalent microorganism group reported (350, 45.3%), followed by *Staphylococcus aureus* (87, 11.3%), *Klebsiella pneumoniae* (72, 9.4%) and *Candida* sp. (49, 6.4%). *Escherichia coli* (28, 3.7%), *Pseudomonas aeruginosa* (27, 3.4%), *Enterococcus faecalis* (24, 3.1%) and *Acinetobacter baumannii* (23, 2.9%) were also frequently identified.

The TTP parameters, including average, min and max time and standard deviation, are presented in table 1 and figure 1.

**Table 1:** Amount, average first time to positivity, min time to positivity and max time to positivity according to species or groups of microorganisms

Species or group of microorganisms	n	Average first TTP		Min TTP	Max TTP
		*X̅*	SD		
CNS ++	350	20	8.40	3	75
CNS +	194	25	11.37	3	82
*Staphylococcus aureus*	87	21	27.54	1	104
*Klebsiella pneumoniae*	72	12	10.84	2	61
*Candida sp.*	49	39	28.42	1	112
*Escherichia coli*	28	10	5.69	1	30
*Pseudomonas aeruginosa*	27	22	14.97	9	68
*Enterococcus faecalis*	24	14	7.33	3	39
*Acinetobacter baumannii*	23	9	3.36	3	19

TTP - time to positivity; SD - standard deviation; CNS ++ - double or more positive coagulase-negative *staphylococci*; CNS + - single positive coagulase-negative *staphylococci*.

**Figure 1:**
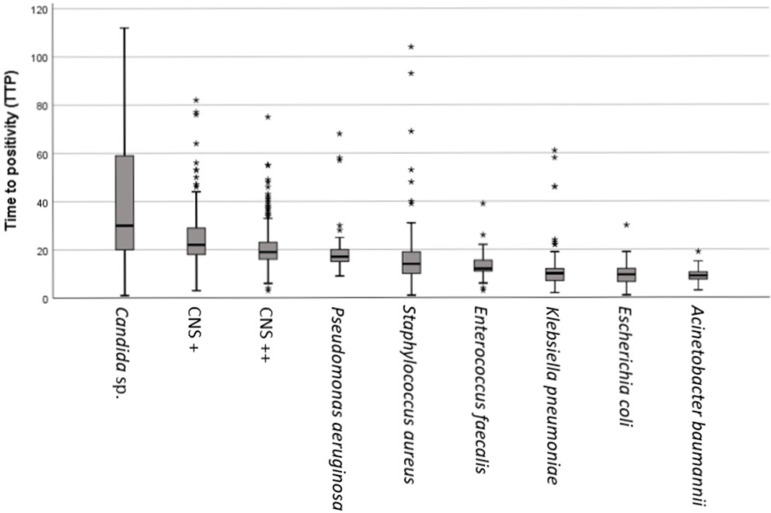
Time to positivity for analyzed species or groups of microorganisms. CNS + - single positive coagulase-negative *staphylococci*; CNS ++ - double or more positive coagulasenegative *staphylococci*.

Regardless of the number of positive cultures, the isolation of some microorganisms, including *Candida* sp., *P. aeruginosa, S. aureus* and *Enterobacteriaceae*, is usually related to high positive predictive values for true BSI.^(7)^ However, due to skin colonization and elevated use of invasive devices such as catheters on ICU subsets, isolation of CNS on blood cultures may be considered a contamination, mainly when no signs or symptoms of bacteremia are described. In this study, TTP of CNS + was significantly higher than TTP of CNS ++ (p < 0.05), TTP of other gram-positive (*Staphylococcus* sp. and *Enterococcus* sp.) (p < 0.05) and TTP of other microorganisms considered noncontaminants (p < 0.05), consistent with previous reports;^(4,8)^ this result suggests TTP as a useful tool to differentiate a true BSI from a contamination.

Time to positivity of different groups of microorganisms (gram-positive, gram-negative and *Candida* sp.) also differed significantly (p < 0.05). Gram-negative microorganisms had lower TTP than fungi and gram-positive microorganisms (p < 0.05). As an exception, TTP of *P. aeruginosa* (22 hours) was longer than TTP of CNS ++ (20 hours), *S. aureus* (21 hours) and *E. faecalis* (14 hours), consistent with previous reports^(4)^. *A. baumannii* had the shortest average TTP (9 hours), and *Candida* sp. had the highest value (39 hours). In the literature, TTP of *Candida* is variable but usually high, with an average minimal TTP values of 27 hours, 35 hours and 41.9 hours.^(9-11)^ The TTP of *A. baumannii* was also consistent with previous reports, which measured TTP values of 10.4 hours and 8.8 hours.^(9,12)^

The distribution of each species or species group within the first 24 hours, 48 hours, 72 hours and > 72 hours of incubation is illustrated in figure 2. With the exception of *Candida*, the number of positive cultures decreased with prolonged incubation. In our study, 75% of pathogens were isolated within 24 hours, 95% within 48 hours and 98% within 72 hours. Ning et al., Pardo et al. and Park et al. previously reported that 95.2%, 97% and 88.3% of all positive cultures, respectively, were detected within 48 hours, and few true BSIs turned positive after 48 hours of incubation; antibiotic de-escalation was recommended after this period for negative cultures.^(4,8,9)^ Our study supports this suggestion, as proper discontinuation of unnecessary antimicrobial therapy reduces hospital expenditure and length of stay and limits selective pressure for antibiotics associated with the development of antimicrobial resistance.^(13)^

**Figure 2:**
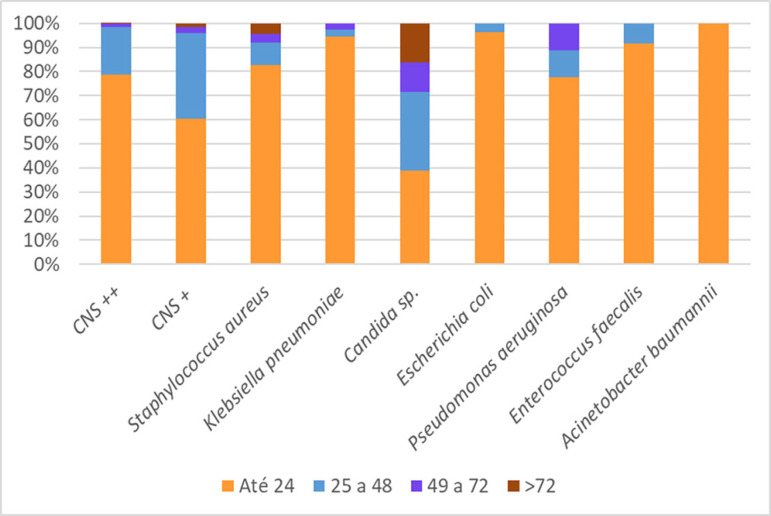
Positive cultures at 24 hours, 48 hours, 72 hours and > 72 hours according to species or groups of microorganisms. CNS ++ - double or more positive coagulase-negative staphylococci; CNS + - single positive coagulase-negative staphylococci.

An association between TTP and clinical outcome in infections has also been suggested. Shorter TTP values reflect a higher circulation of microorganisms, and the microbial load may be associated with higher mortality rates. In our study, this association was significant for *Candida* sp. (p < 0.05). Patient mortality was higher when the culture for *Candida* was positive before 37 hours (area under the curve - AUC, 0.733; sensitivity, 83%; specificity, 60%; p = 0.005), indicating that TTP can be used as a predictor for mortality in patients with candidemia, consistent with previous studies.^(10)^ According to Nunes et al., no statistically significant difference was observed between patients with early TTP for *Candida* (< 36 hours) and late TTP (> 36 hours).^(11)^ However, Kim et al. associated mortality for *Candida* sp. with a TTP of < 24 hours.^(10)^ No correlation was found for bacterial species in our study.

This study highlights TTP as a useful tool to distinguish a contaminant from a true BSI infection and can also be used as a predictor of mortality in infections caused by *Candida* sp. In addition, since 95% of cultures were positive for up to 48 hours of incubation, this time can be used for de-escalation of antimicrobials in patients with suspected bacteremia with negative culture results, since rare BSI are evident after this incubation period.

## References

[1] Russotto V, Cortegiani A, Graziano G, Saporito L, Raineri SM, Mammina C (2015). Bloodstream infections in intensive care unit patients: distribution and antibiotic resistance of bacteria. Infect Drug Resist.

[2] Bassetti M, Righi E, Carnelutti A (2016). Bloodstream infections in the intensive care unit. Virulence.

[3] Silva E, Dalfior Junior L, Fernandes HS, Moreno R, Vincent JL (2012). Prevalence and outcomes of infections in Brazilian ICUs: a subanalysis of EPIC II study. Rev Bras Ter Intensiva.

[4] Ning Y, Hu R, Yao G, Bo S (2016). Time to positivity of blood culture and its prognostic value in bloodstream infection. Eur J Clin Microbiol Infect Dis.

[5] Araujo MR (2012). Hemocultura: recomendações de coleta, processamento e interpretação dos resultados. J Infect Control.

[6] Murray PR, Rosenthal KS, Pfaller MA (2009). Microbiologia Médica.

[7] Weinstein MP, Towns ML, Quartey SM, Mirrett S, Reimer LG, Parmigiani G (1997). The clinical significance of positive blood cultures in the 1990s: a prospective comprehensive evaluation of the microbiology, epidemiology, and outcome of bacteremia and fungemia in adults. Clin Infect Dis.

[8] Pardo J, Klinker KP, Borgert SJ, Trikha G, Rand KH, Ramphal R (2014). Time to positivity of blood cultures supports antibiotic de-escalation at 48 hours. Ann Pharmacother.

[9] Park SH, Shim H, Yoon NS, Kim MN (2010). Clinical relevance of time-to-positivity in BACTEC9240 blood culture system. Korean J Lab Med..

[10] Kim SH, Yoon YK, Kim MJ, Sohn JW (2013). Clinical impact of time to positivity for Candida species on mortality in patients with candidaemia. J Antimicrob Chemother.

[11] Nunes CZ, Marra AR, Edmond MB, da Silva Victor E., Pereira CA (2013). Time to blood culture positivity as a predictor of clinical outcome in patients with Candida albicans bloodstream infection. BMC Infect Dis.

[12] Lai CC, Wang CY, Liu WL, Cheng A, Lee YC, Huang YT (2011). Time to blood culture positivity as a predictor of drug resistance in Acinetobacter baumannii complex bacteremia. J Infect.

[13] Paterson DL, Rice LB (2003). Empirical antibiotic choice for the seriously ill patient: are minimization of selection of resistant organisms and maximization of individual outcome mutually exclusive?. Clin Infect Dis.

